# Antibiofilm Activity of Sundew Species against Multidrug-Resistant *Escherichia coli* Strains

**DOI:** 10.3390/ijms232213720

**Published:** 2022-11-08

**Authors:** Sandy Gerschler, Sebastian Guenther, Christian Schulze

**Affiliations:** Pharmaceutical Biology, Institute of Pharmacy, University of Greifswald, Friedrich-Ludwig-Jahn-Straße 17, 17489 Greifswald, Germany

**Keywords:** naphthoquinones, flavonoids, anti-biofilm, *E. coli*, *Drosera rotundifolia*, *Drosera intermedia*, biological activities

## Abstract

Species of the genus *Drosera*, known for carnivorous plants, such as sundew, have been traditionally used for centuries as medicinal plants. Efficacy-determining compounds are naphthoquinones and flavonoids. Flavonoids possess a broad spectrum of bioactive properties, including biofilm inhibitory activity. Biofilms render antibiotics ineffective, contributing to the current rise in antimicrobial resistance. In this study, the biofilm inhibitory activity of two European sundew species (*Drosera rotundifolia* and *Drosera intermedia*) grown agriculturally in Germany and four commercial sundew products (declared as *Drosera longifolia*, *Drosera* sp. and Drosera planta trit.) against three multidrug-resistant *Escherichia coli* strains was tested. The aim of the study was to comparatively investigate the biofilm inhibitory potential of sundew species extracts grown locally in northern Germany and commercial sundew products. The minimum biofilm inhibitory concentration of the European sundew species was approx. 35 µg mL^−1^. In comparison, commercial sundew products ranged in concentration from 75 to 140 µg mL^−1^. Additionally, individual compounds isolated from European sundew were tested. Among these compounds, biofilm inhibitory activity was determined for four of the eight substances, with 2″-O-galloyl hyperoside standing out for its activity (38 µg mL^−1^). The whole plant extracts of *Drosera rotundifolia* and *Drosera intermedia* proved to be more effective than the commercial products and the single compounds in its biofilm inhibition activity against *Escherichia coli* strains. Sundew extracts may serve as a potential therapeutic approach for targeting biofilm production.

## 1. Introduction

*Drosera*, the second largest genus of carnivorous plants, includes over 200 species. Typical of the genus are the small, tentacle-like outgrowths of the leaves, which are covered with adhesive glands and are used to catch and digest insects. The plant uses the insects as a source of nitrogen, making it ideally adapted for life in nutrient-poor areas, such as peatlands. Sundews are distributed worldwide, from North America, through Europe and Africa, to Asia [1]. European species include the round-leaved sundew, *Drosera* (*D.*) *rotundifolia*, and the oblong-leaved sundew *D*. *intermedia*. In Central Europe, the plants have traditionally been used to treat spasmodic respiratory diseases [2]. Despite their recognized efficacy, sundew is not found in rational phytotherapy today. The reason is that sundew is a protected plant, and thus may no longer be collected in most European countries [3]. As a consequence, commercial sundew biomass has been imported to Europe from other regions of origin, such as East Africa and Madagascar [4,5,6]. However, non-European *Drosera* species have significantly lower concentrations of efficacy-determining natural compounds [7]. Recent large-scale cultivation experiments of *Drosera* species in combination with peat mosses in northern Germany have, however, shown that agricultural cultivation of European species is now a possibility [8]. The Paris Agreement implies the rewetting of 500,000 km^2^ of drained peatlands worldwide in a period from 2050 to 2070. For Germany, about 50,000 ha per year would have to be rewetted to achieve this goal [9]. As sundew can be grown and harvested on parts of these wetlands, its reintroduction into the pharmaceutical market presents a commercial opportunity for wetland farm owners. However, for a rational phytotherapy, intensive characterization of the chemical compounds of the herbal drug is required. In addition, this also offers the potential to expand its use to new indications

The antimicrobial-active naphthoquinones, such as plumbagin and 7-methyl juglone, have long been known to be responsible for the effects of the drug *Drosera herba* [10]. In vitro studies have shown that flavonoids and ellagic acid derivatives contribute to anti-inflammatory and anti-spasmodic effects [4,11,12]. Flavonoids possess a versatile bioactivity, such as antioxidant, cardioprotective or biofilm inhibitory activity [13]. Multi-layered cellular coatings, so-called biofilms, are often surrounded by a cover of polysaccharides and proteins, which act as a diffusion barrier to provide greater resistance to physical and chemical stimuli. In medicine, biofilms are a problem because biofilm-forming pathogens require antibiotic concentrations up to 1000-fold higher than in their planktonic state [14,15]. If, in addition, multidrug-resistant pathogens are involved in the infection process, this can cause increased treatment times or even unsuccessful treatments. Particularly alarming are ESBL (extended-spectrum beta-lactamase)-producing Gram-negative enterobacteria, such as *Escherichia* (*E.) coli*, which are involved in urinary tract infections (UTI), for example [16]. It is estimated that approximately 150 million people worldwide are affected each year, the majority of whom are women. Approximately half of all women will suffer from a UTI at least once during their lifetime [17,18]. The most common pathogen in a UTI is *E. coli*, especially zoonotic multidrug-resistant high-risk clonal lineages, such as ST131 and ST648, which often combine resistance with virulence features, such as biofilm formation. These bacteria strains present a growing burden on human and animal health [19]. New insights into virulence factors, such as biofilm formation, offer innovative therapeutic options and may consequently reduce the prescription frequency of antibiotics, and thus have a positive effect on the resistance situation [20]. *E. coli* as a model organism can help to understand such mechanisms, and thus identify targets for biologically active substances. The investigation of the antibacterial naphthoquinones and potentially biofilm-inhibiting flavonoids contained in sundew could represent an effective combination for the therapy of multidrug-resistant *E. coli* strains, and thus reveal a further indication area.

The aim of this study was to investigate the biofilm inhibitory effect of two European sundew species (*D. rotundifolia* and *D. intermedia*) grown in northern Germany and to compare the effect with commercially available sundew products. In addition, the effect of individual compounds of the sundew extracts was analyzed in order to draw conclusions on efficacy-determining ingredients.

## 2. Results and Discussion

### 2.1. Identification and Quantification of Flavonoids and Naphthoquinones

**Figure 1:**
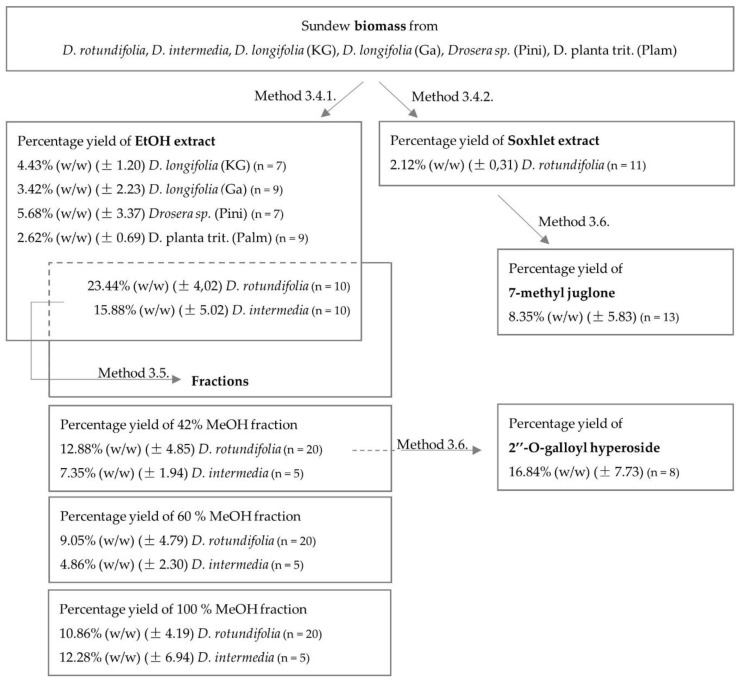
An overview of the percentage yield of extractions, fractionation and isolated compounds.

Flavonoids and naphthoquinones belong to the pharmaceutically interesting secondary plant compounds. To investigate how the naphthoquinone and flavonoid content differed between sundew species grown in Germany and commercially available sundew biomass, ultrasound extracts were prepared and quantified using European Pharmacopoeia methods and an HPLC method. Fractions were obtained from the extracts in which compoundswere enriched and flavonoids were separated from naphthoquinones. The aim of the fractionation was to compare the microbiological effect of the flavonoids with that of the naphthoquinones and to obtain information on the efficacy of certain compoundsby enriching them. In addition, the fractionation served as a purification step for the isolation of the non-commercially available flavonoid 2″-O-galloyl hyperoside. For the isolation of the naphthoquinone 7-methyl juglone, which is also not commercially available, a Soxhlet extraction with n-hexane was performed because the yield of 7-methyl juglone was too low with ultrasonic extraction. An overview of the extraction, fractionation and isolation carried out is shown in Figure 1. Comparing the average extract yield of the commercial sundew biomass (4% *w*/*w*) with the average biomass cultivated in Germany (20% *w*/*w*), the extract yield of the sundew material cultivated in Germany is about five times higher. The comparison of the yield of the species cultivated in Germany with each other shows that the extract yield of *D. rotundifolia* is about 2/3 higher than that of *D. intermedia*. The fraction yield of the 42% and 60% MeOH fraction is also observed to be higher for *D. rotundifolia* than for *D. intermedia*. Except for the 100% fraction, the fraction yield is lower for *D. rotundifolia* than for *D. intermedia*. This could be due to the composition of flavonoids and naphthoquinones amounts. The 42% and 60% fractions contain predominantly flavonoids, and these are mainly found in *D. rotundifolia*. In contrast, the 100% fraction contains naphthoquinones, of which there are more in *D. intermedia* than in *D. rotundifolia*. The yields of the isolations show that about 17% (±8%) 2″-O-galloyl hyperoside is isolated from the 42% fraction of *D. rotundifolia* and 8% (±6%) 7-methyl juglone could be isolated from the soxhlet extract of *D. rotundifolia*.

#### 2.1.1. Identification of 7-Methyl Juglone

**Figure 2:**
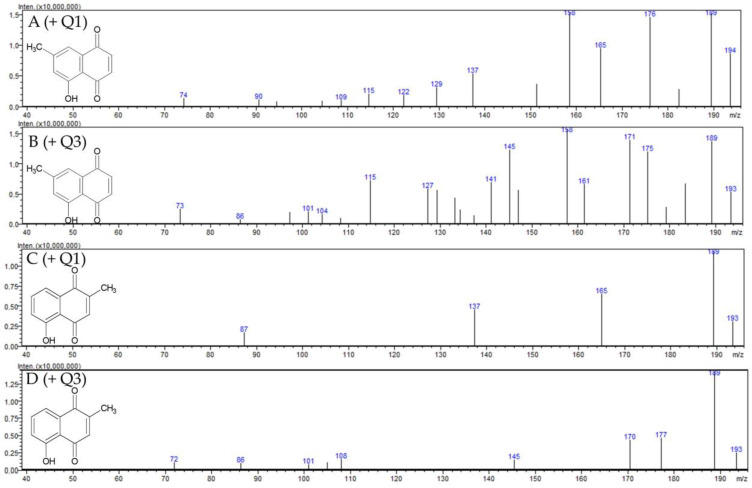
Mass spectra (LC–ESI–MS-MS) of 7-methyl juglone and plumbagin: (**A**) positive ESI-quadrupole (Q1) mass spectra of 7-methyl juglone; (**B**) positive ESI-quadrupole (Q3) mass spectra of 7-methyl jugline; (**C**) positive ESI-quadrupole (Q1) mass spectra of plumbagin; (**D**) positive ESI-quadrupole (Q3) mass spectra of plumbagin.

The composition of naphthoquinones differs in the *Drosera* species *D. rotundifolia* and *D. intermedia*. Whereas *D rotundifolia* contains mainly 7-methyl juglone, *D. intermedia* primarily includes plumbagin [21]. Therefore, the extract of *D. rotundifolia* was used to isolate the 7-methyl juglone, which is not commercially available. For identification, a mass scan and multiple reaction monitoring (MRM) optimization of 7-methyl juglone, comparatively to the commercially available plumbagin (5-hydroxy-1,4-napthoquinone), was performed. These compounds are constitutional isomers that differ only in the position of a methyl group. Therefore, the molecular peak *m*/*z* 189 is found in both scans (see Figure 2). The MRM transitions 188.9 → 161.15, 188.9 → 114.95 and 188.9 → 105.10 were identified in both compounds (see Table 1). The pseudomolecular ion *m*/*z* 161 of plumbagin was also described for identification in the study by Medic et al. [22]. The molecular peak and the same MRM transitions indicate the structural similarity of both substances. The different MRM transitions are significant for the distinction of the isolated 7-methyl juglone from plumbagin; 188.9 → 157.05, 188.9 → 77.05 and 188.9 → 114.95 are among the five most abundant transitions for the 7-methyl juglone and 188.9 → 121.05, 188.9 → 144.95, as well as 188.9 → 65.20 for plumbagin (see Table 1).

#### 2.1.2. Identification of 2″-O-Galloyl Hyperoside

**Figure 3:**
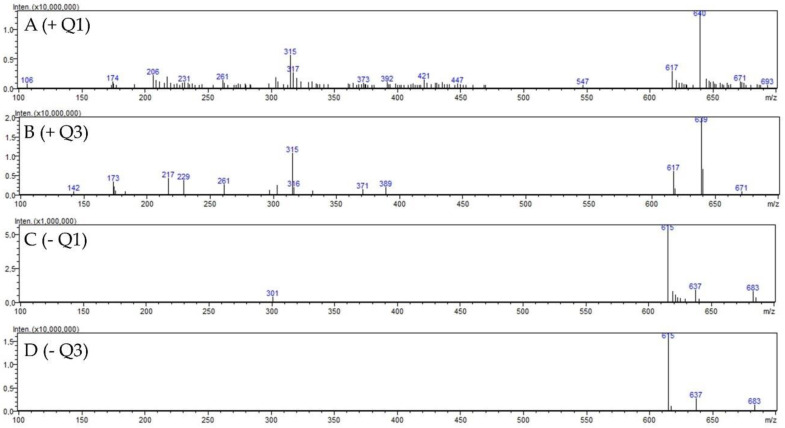
Mass spectra (LC–ESI–MS) of 2″-O-galloyl hyperoside: (**A**) positive ESI-Quadrupole (Q1) mass spectra of 2″-O-galloyl hyperoside; (**B**) positive ESI-quadrupole (Q3) mass spectra of 2″-O-galloyl hyperoside; (**C**) negative ESI-quadrupole (Q1) mass spectra of 2″-O-galloyl hyperoside; (**D**) negative ESI-quadrupole (Q3) mass spectra of 2″-O-galloyl hyperoside.

**Figure 4:**
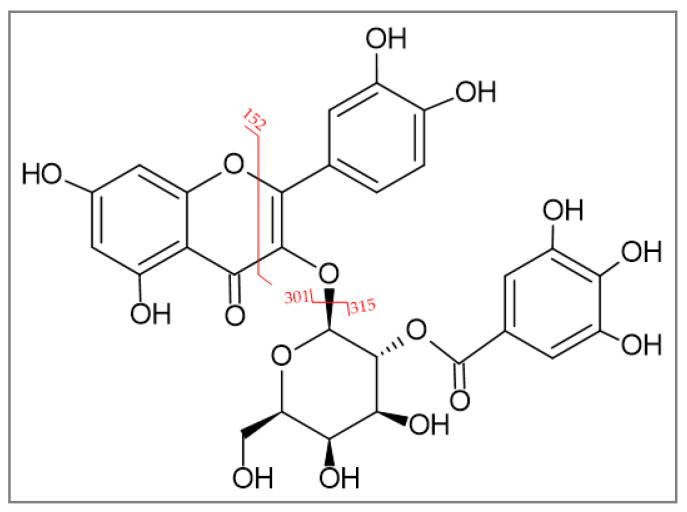
Different cleavage sites of 2″-O-galloyl hyperoside.

For the isolation of 2″-O-galloyl hyperoside, the 42% fraction of *D. rotundifolia* was used since significantly more 2″-O-galloyl hyperoside was present in *D. rotundifolia* than in *D. intermedia*. For this purpose, 2″-O-galloyl hyperoside was enriched from the EtOH extract of *D. rotundifolia* by solid phase extraction in the 42% fraction and then isolated from the fraction by size exclusion extraction. Mass scanning and MRM optimization of the 2″-O-galloyl hyperoside were performed for identification. The molecular peak *m*/*z* 617 in the positive scan and *m*/*z* 615 in the negative scan shown in Figure 3. The pseudomolecular ions *m*/*z* 151, 301, and 313 are characteristic of 2″-O-galloyl hyperoside and have been used for identification in other studies as well (see Table 2) [7,23]. Different cleavage sites of 2′′-O-galloyl hyperoside are shown in Figure 4.

#### 2.1.3. Quantification of Extracts and Fractions

**Figure 5:**
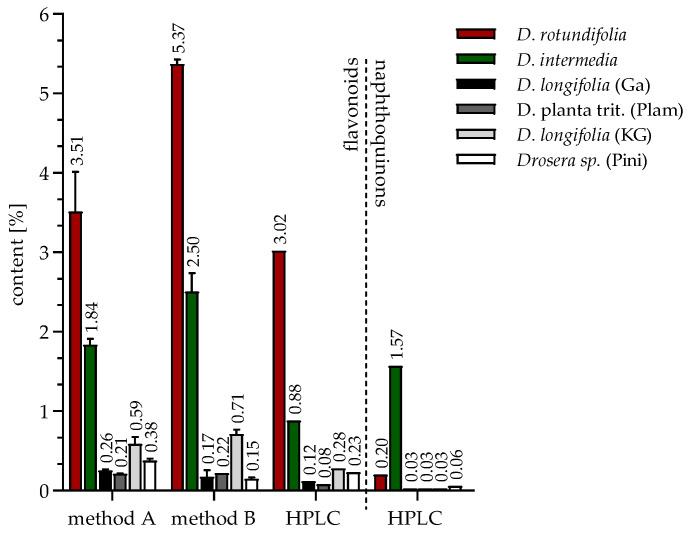
Flavonoid and naphthoquinone content of different *Drosera* species analyzed by three methods: HPLC (detected flavonoids: 2″-O-galloyl hyperoside, hyperoside, isoquercetin; quercetin and detected naphthoquinones: 7-methyl juglone, plumbagin) and two European Pharmacopoeia (Ph.Eur. 9.7, 01/2017:1174) content determination methods from the monographs, birch leaves (method A) and hawthorn leaves with flowers (method B); values indicate percentage of dry plant weight.

Due to its high flavonoid content [14], sundew has the potential to have a biofilm inhibitory effect. Therefore, in the first part, a comparative study of the biofilm inhibitory effect of two European sundew species (*D. rotundifolia* and *D. intermedia*) and commercially available sundew biomass was performed to evaluate their usefulness as an agent against biofilm formation in multidrug-resistant *E. coli*. In the second part, the effects of potential active compounds of the sundew extract were analyzed. For this purpose, EtOH extracts were prepared and compounds were enriched into fractions or single substances isolated from sundew extracts. The flavonoid content was analyzed with two European Pharmacopoeia (PhEur) methods and a HPLC method (Zehl et al., modified) [7], which allows the quantification of four flavonoids (hyperoside, isoquercetin, quercetin and 2″-O-galloyl hyperoside), two naphthoquinone compounds (plumbagin and 7-methyl juglone), gallic acid and ellagic acid. The data analyzed are summarized in the following Figure 5. The following values indicate the percentage of dry plant weight. Among the sundew species studied, *D. rotundifolia* contained the highest flavonoid content of 5.37% (method B), 3.51% (method A) and 3.02% (HPLC). *D. intermedia* contained a much lower content of 2.50% (method B), 1.84% (method A), and 0.88% (HPLC). In comparison, commercial sundew biomasses have an average content of 0.31% (method B), 0.69% (method A) and 0.18% (HPLC), which are significantly lower than the flavonoid content of *D. rotundifolia* and *D. intermedia*. The differences in the flavonoid contents in the two different European Pharmacopoeia methods used can be explained by the different ways of color complex formation. Whereas the aluminum chelate complex method (method A) predominantly determines flavonoid glycosides that are O-glycosidically linked, the boric acid oxalic acid method (method B) detects both C- and O-glycosidically linked flavonoids. The flavonoids hyperoside, quercetin, isoquercetin and 2″-O-galloyl hyperoside quantified by HPLC analysis account for more than half of the total flavonoid content, and thus appear to be among the major flavonoids in the tested sundew extracts. The examination of the naphthoquinones plumbagin and 7-methyl juglone, quantified by HPLC, revealed that the extract of *D. intermedia* has eight times higher content (1.57%) than *D. rotundifolia* (0.20%) and has a content about 25 times higher than commercial biomasses (0.03–0.06%). In a study by Zehl et al. [7], 13 compounds were identified and quantified from four *Drosera* species using an LC-MS method, including the flavonoids hyperoside, isoquercetin, quercetin, and 2″-O-galloyl hyperoside. Again, *D. rotundifolia* was found to have a significantly higher flavonoid content, compared to *D. intermedia* [7]. It is interesting to note that in this study, qualitative flavonoid composition was comparable in different sundew species, such as *D. rotundifolia*, *D. tokaiensis*, and *D. spatulata*, but great variations in the quantity of the compounds were found. Some sundew species contain flavonoid content above 6% based on dry weight of plant material, while others contain hardly any flavonoids [2]. Since flavonoids are believed to be important for therapeutic effects and flavonoid content varies widely among sundew species, the use of *Drosera* sp. on the basis of genus only, rather than species, is not appropriate for the preparation of the drug *Drosera herba*. The European species, round-leaved sundew (*D. rotundifolia*), with flavonoid content up to 6%, has been previously used as a starting material for obtaining the medicinal drug *Drosera herba* [24]. Based on our results, this seems reasonable as its flavonoid contents are much higher than in the other European species, *D. intermedia,* and also than in the commercially obtained sundew biomass declared as *D. longifolia* and D. planta trit. One problem with products containing *D. longifolia* is that they are often mislabeled, as in some cases this species is used as a synonym for *D. madagascariensis*, which has significantly lower contents of flavonoids and naphthoquinones [2,7,25]. This leads to the assumption that the examined sundew products might contain *D. madagascariensis* and not *D. longifolia* as declared. Therefore, only *D. rotundifolia* should be used for the production of phytopharmaceuticals, and this should be ensured by chemical analytical quality standards.

**Figure 6:**
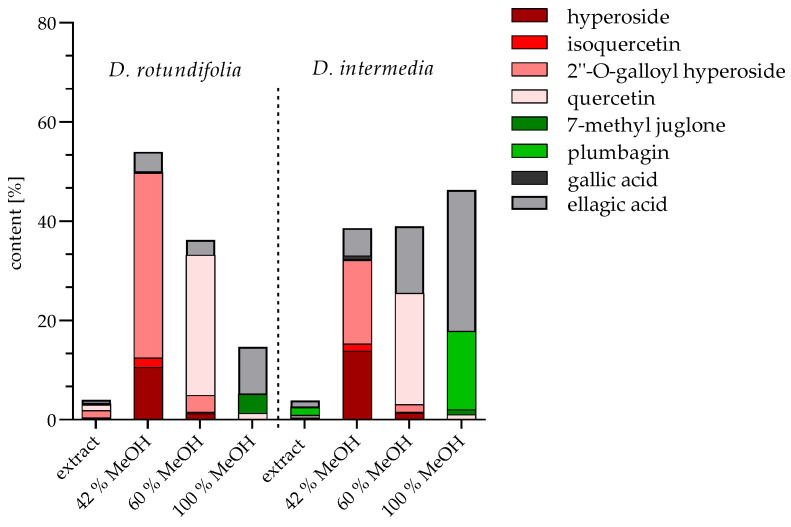
Content of flavonoids (2″-O-galloyl hyperoside, hyperoside, isoquercetin, quercetin), naphthoquinones (7-methyl juglone, plumbagin), ellagic acid and gallic acid per dry fraction weight of *D. rotundifolia* and *D. intermedia* stated in percent; EtOH extracts are given as percentages of the dry weight of the extracts; all measurements were determined by HPLC.

In order to obtain preliminary indications of which compounds investigated in this study are responsible for a biofilm inhibitory effect, fractions were obtained from the EtOH whole plant extracts by solid phase extraction chromatography and quantified by HPLC method. The composition of these fractions is shown in Figure 6. Compound groups in the extracts were separated into different fractions according to their polarity with increasing MeOH concentration. Three fractions were prepared from *D. rotundifolia* and *D. intermedia*, quantified by HPLC, and the values indicate percentage of dry fraction weight. The 42% MeOH fraction contains mainly 2″-O-galloyl hyperoside (37.27% for *D. rotundifolia*; 16.85% for *D. intermedia*), besides hyperoside (10.53% for *D. rotundifolia*; 13.88% for *D. intermedia*), ellagic acid (3.96% for *D. rotundifolia*; 5.61% for *D. intermedia*), isoquercetin (1.93% for *D. rotundifolia*; 1.42% for *D. intermedia*), and gallic acid (0.25% for *D. rotundifolia*; 0.96% for *D. intermedia*). The composition of 60% MeOH fraction mainly includes quercetin (28.25% for *D. rotundifolia*; 22.42% for *D. intermedia*) and hyperoside (1.25% for *D. rotundifolia*; 1.40% for *D. intermedia*). The 60% MeOH fraction also contains a small amount of 2″-O-galloyl hyperoside (3.44% for *D. rotundifolia*; 1.46% for *D. intermedia*). In addition, the 60% MeOH fraction of *D. intermedia* include a high proportion of ellagic acid (13.52%), in contrast to the 60% MeOH fraction of *D. rotundifolia* (3.01%). The 100% MeOH fraction consists mainly of ellagic acid (9.40% for *D. rotundifolia*; 28.42% for *D. intermedia*) and naphthoquinones, plumbagin in *D. intermedia* (15.86%) and 7-methyl juglone in *D. rotundifolia* (3.89%). Furthermore, quercetin (1.28%) is also present in 100% MeOH fraction for *D. rotundifolia*. Fractionation allowed the rough subdivision into a flavonoid glycoside fraction (42% MeOH fraction); a quercetin fraction (fraction 60% MeOH), which also contains plumbagin in *D. intermedia*; and a naphthoquinone fraction (100% MeOH fraction) with additional quercetin in *D. rotundifolia*. In the further aspects of the work, it was investigated whether the biofilm-inhibiting effect differs within the fractions, and thus provides indications of efficacy-determining ingredients.

### 2.2. Microbiological Testing

In addition to naphthoquinones, sundew contains a high level of flavonoids, another group of compounds of pharmaceutical interest, as flavonoids have a broad spectrum of bioactive effects. Therefore, it makes sense to investigate other possible indications [26]. A study on biofilm formation of *E. coli* has shown that flavonoids, such as quercetin and myricetin, are able to reduce the extracellular matrix by inhibiting the formation of amyloid curli fibers [27]. The aim of this study was to investigate the effect of flavonoid containing sundew extracts, fractions and single substances on the formation of the extracellular matrix (cellulose and curli) of three multidrug-resistant *E. coli* strains.

#### 2.2.1. Minimum Inhibitory Concentration (MIC)

To ensure that the biofilm inhibitory effect of the extracts, fractions and compounds was not due to a general antimicrobial effect, the minimum inhibitory concentration was determined for all samples. Supplementary Table S1 shows that the EtOH extracts, the 42% MeOH, 60% MeOH, and 100% MeOH fractions of *D. rotundifolia*, did not show antibacterial properties against the tested multidrug-resistant *E. coli* strains in the tested concentration range of 25–1000 µg mL^−1^. The same was the case for the 42% MeOH and 60% MeOH fractions of *D. intermedia* and the compounds 2″-O-galloyl hyperoside, hyperoside, isoquercetin, quercetin, gallic acid and ellagic acid, as well as the EtOH extracts of the commercial sundew biomasses. Although the flavonoid aglycons showed no activity, the naphthoquinones plumbagin and 7-methyl juglone possessed antibacterial activity against all three *E. coli* strains in the range of 94 to 250 µg mL^−1^. Interestingly, fraction 100% MeOH of *D. intermedia* presented antibacterial activity against the tested *E. coli* strains in the range of 533 to 700 µg mL^−1^. In 100% MeOH fraction, the percentage of plumbagin and 7-methyl juglone is about 17%. Assuming that only these two compounds are responsible for the antibacterial effect and that the antibacterial effect is linear with the concentration in the tested range, the MIC should be significantly higher than the values for plumbagin and 7-methyl juglone. The MIC of the 100% MeOH fraction ranged from 500 to 700 µg mL^−1^. In these fractions, the naphthoquinones plumbagin and 7-methyl juglone were enriched. Depending on the *Drosera* species used, the fraction contained a range of plumbagin and 7-methyl juglone from 4% for *D. rotundifolia* to 17% for *D. intermedia* (see Figure 6). Naphthoquinones, such as plumbagin, are known to have antimicrobial activity [28], which was also demonstrated in this study (plumbagin 100–200 µg mL^−1^ and 7-methyl juglone 200–300 mg mL^−1^, see Supplementary Table S1). However, it is noticeable that the observed inhibitory concentrations for pure compounds are relatively high and show a large scatter around the mean value. Nevertheless, the determined inhibitory concentrations may give an indication that the naphthoquinones contained in the 100% MeOH fraction may be responsible for the antimicrobial activity of this fraction. The fact that plumbagin, 7-methyl juglone and the 100% MeOH fraction possess antimicrobial activity is important for the interpretation of the biofilm inhibitory activity of the fractions and individual substances investigated in this study. It should also be taken into account that the 100% MeOH fraction is a mixture of substances, which could be the reason why the concentration for the antimicrobial activity of the fraction is higher than the inhibitory concentrations of plumbagin and 7-methyl juglone. In addition, it is possible that other components may influence the effect of 7-methyl juglone or plumbagin by interacting with the substances. In general, it can be said that naphthoquinones, such as plumbagin, have antibacterial activity [29], in addition to their antifungal [30], anticancer [31] and antimutagenic [32] effects. Naphthoquinones are cyclic redox systems and can generate reactive oxygen species to induce oxidative stress [33,34]. Studies on the antibacterial mechanism of the action of plumbagin against *E. coli* have shown that plumbagin alters lactose carriers to the extent that there is a loss of galactoside binding ability. This affects energy metabolism for some secondary transport systems [35]. In addition, the inhibition of NADH dehydrogenase, a primary respiratory dehydrogenase, in the glucose-containing medium of *E. coli* by plumbagin has been postulated [36]. A 2011 review summarized many in vitro studies postulating an antibacterial effect of flavonoids [37]. Studies of the antibacterial properties of kaempferol, quercetin, myricetin, rutin, naringin, and formononetin from various plants in terms of their antimicrobial activity against various bacteria and fungi showed effects, but none of the compounds had an inhibitory effect against *E. coli* [38,39]. Another study investigated plant extracts and phenolic substances, including quercetin, against various germs by agar diffusion assay. Quercetin showed only mild antimicrobial activity against *E. coli* [40]. It is possible that the effect of quercetin and other flavonoids against *E. coli* is not shown by an antibacterial effect but by biofilm inhibitory properties. In conclusion, the extracts of *D. rotundifolia*, *D. intermedia* and the commercial biomass of *D. longifolia* and D. planta trit., as well as the fractions of *D. rotundifolia* and *D. intermedia* did not show antibacterial activity in the tested concentration range for biofilm inhibition. It concludes that the investigated inhibitory effect is based on the specific inhibition of biofilm formation.

#### 2.2.2. Minimum Biofilm Inhibitory Concentration (MBIC)

**Figure 7:**
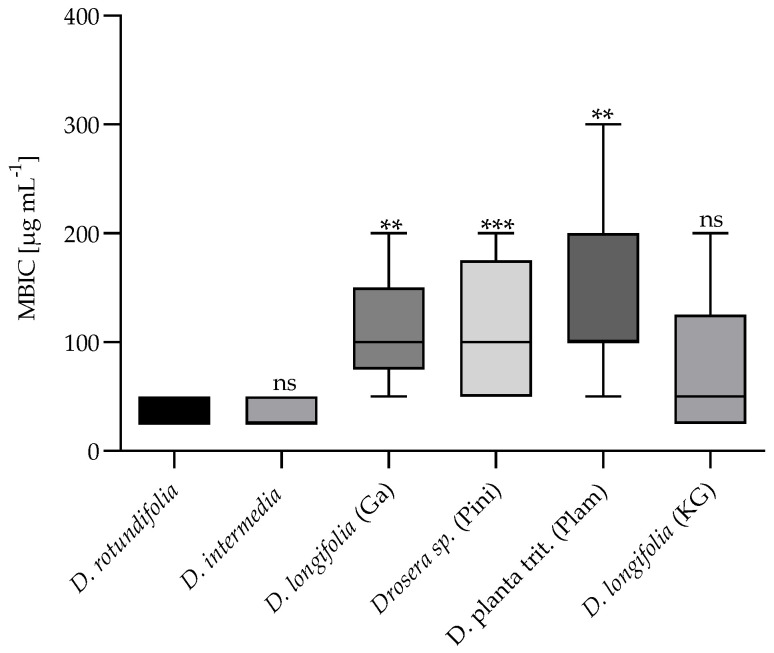
Minimum biofilm inhibitory concentration (MBIC) of six sundew samples against three multi-drug-resistant *E. coli* strains (PBIO729, PBIO730, PBIO1986). To evaluate whether the commercial sundew biomasses and *D. intermedia* had the same biofilm inhibitory efficacy as the species *D. rotundifolia*, the MBICs of the samples *D. intermedia*, *D. longifolia* (GA), *Drosera* sp. (Pini), D. planta trit. (Plam) and *D. longifolia* (KG) were compared with the sample *D. rotundifolia*; a significant difference in MBIC to D. rotundifolia indicates a reduced efficacy [n = 3/*E.coli* sp.]; data were analyzed by Kruskal–Wallis test; ns: *p* ≥ 0.05, **: *p* ≤ 0.0021, ***: *p* ≤ 0.0002.

**Figure 8:**
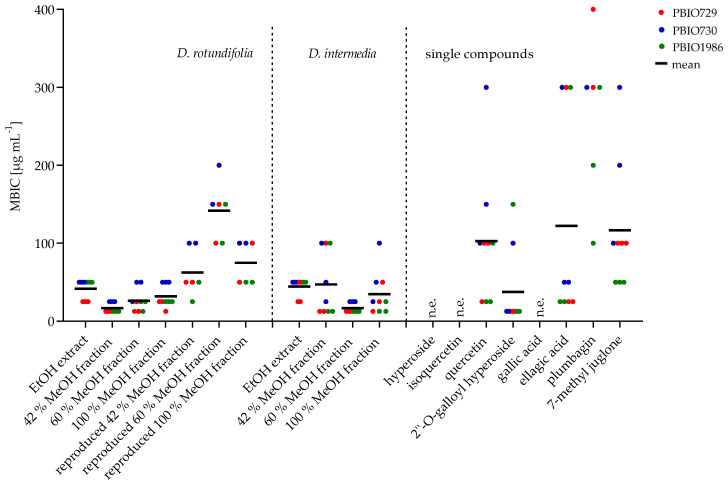
Minimum biofilm inhibitory concentration (MBIC) of *D. rotundifolia* (EtOH extract, MeOH fractions, reproduced MeOH fractions), *D. intermedia* (EtOH extract, MeOH fractions) and pure compounds (hyperoside, isoquercetin, 2″-O-galloyl hyperoside, gallic acid, ellagic acid, plumbagin, 7-methyl juglone) against three *E. coli* strains (PBIO729, PBIO730, PBIO1986).

Biofilm inhibition assay was performed using a long-term colony assay in which the matrix component, cellulose and curli fimbriae were stained with Congo red and Coomassie blue. The inhibition of matrix formation was analyzed visually based on their decolorization at concentrations ranging from 12.5 to 400 µg mL^−1^. The EtOH extracts and fractions of the studied *Drosera* species and four of the eight compounds of the sundew extract presented biofilm inhibitory effects in all *E. coli* strains. This indicates that the biofilm inhibitory effect of the extracts, fractions and compounds are not strain-specific. Figure 7 shows the results of the comparative study of the biofilm inhibitory effect of the sundew species cultivated in Germany with the commercially available sundew biomasses. The extracts of *D. rotundifolia* and *D. intermedia* grown in Germany showed a similar biofilm inhibitory effect at a concentration of 25–50 µg mL^−1^. To achieve an inhibitory effect of biofilm formation by the EtOH extracts of the commercial sundew biomasses, approximately double to triple the amount of extract was required on average. The 42%, 60% and 100% MeOH fractions of *D. rotundifolia* and 60% MeOH fraction of *D. intermedia* showed inhibitory activity in a range of 12.5–50 µg mL^−1^ for all *E. coli* strains. The 42% and 100% MeOH fractions of *D. intermedia* possess a stronger effect for strains PBIO730 and PBIO1986 (12.5–25 µg mL^−1^) than for PBIO729 (58.3–75 µg mL^−1^). Examination of the individual compounds revealed that ellagic acid possessed biofilm inhibitory properties at a concentration of approximately 120–135 µg mL^−1^. Of the four flavonoids tested (hyperoside, quercetin, 2″-O-galloyl hyperoside and isoquercetin), only quercetin (75–183.33 µg mL^−1^) and 2″-O-galloyl hyperoside (12.5–58.33 µg mL^−1^) had a biofilm inhibitory effect. Compared to flavonoids, the naphthoquinones plumbagin and 7-methyl juglone showed significantly lower biofilm inhibitory effects against the tested *E. coli* strains. Whereas plumbagin was only active in a concentration of 200 µg mL^−1^ against one *E. coli* strain (PBIO1986), 7-methyl juglone showed inhibitory effects against all strains tested (50–200 µg mL^−1^). These two naphthoquinones are constitutional isomers. They differ only in the position of a methyl group. It would be conceivable that due to the altered spatial arrangement, 7-methyl juglone can no longer bind to a potential target, and thus further reaction cascades fail to occur. No significant difference in the inhibitory activity of the tested fractions could be investigated (see Figure 8). A possible explanation could be the composition of the fractions. Figure 6 summarizes the results of the quantitative analysis. The main component of 42% MeOH fraction is 2″-O-galloyl hyperoside, in addition to hyperoside. In 60% MeOH fraction quercetin was dominating in *D. rotundifolia and* in *D. intermedia*. In 100% MeOH fraction ellagic acid was the main component, as well as plumbagin in *D. intermedia* or 7-methyl juglone in *D. rotundifolia*. These are single substances; all of which showed activity, thus, each fraction has biofilm inhibitory compounds. It is also conceivable that the fractions contain unknown substances that have a biofilm-inhibiting effect. Therefore, the fractions were replicated using reference substances and isolated compounds and evaluated for their biofilm inhibitory activity. The unknown content of the fractions was replaced by xylitol. It was determined beforehand that xylitol has no influence on the biofilm formation of the *E. coli* strains used. The results are shown in Figure 8. The replicated 42% MeOH fraction of *D. rotundifolia* was effective in a concentration range of 58–67 µg mL^−1^, 60% MeOH fraction from 133 to 150 µg mL^−1^ and 100% MeOH fraction 83–67 µg mL^−1^. Comparing the values with the original fractions, these are in a concentration range from 12.5 to 50 µg mL^−1^. The MBIC of the reconstituted 42% MeOH fraction is only slightly higher than the original fraction. It can be assumed that the 2″-O-galloyl hyperoside is decisive for the activity of this fraction. In the case of 60% MeOH and 100% MeOH fractions, the reconstituted fractions require double to triple concentration for an effect, which may be an indication of unknown efficacy-determining or efficacy-increasing accompanying substances. In contrast, the inhibitory effect of the single substances varied among the *E. coli* strains. Compared to the single substances, the MBIC of the extracts and fractions is much lower. An exception is 2″-O-galloyl hyperoside, which shows a biofilm inhibitory effect against PBIO730 already at 12.5 µg. These results suggest that the effect of the tested single substances within an extract or fraction could be enhanced by synergistic effects. Herbal multi-substance mixtures, such as extracts, seem to be more useful for therapeutic application than isolated single substances, as they might have a broader spectrum of activity.

The biofilm assay performed in this study was used to analyze the inhibitory effect of substances or substance combinations on the formation of curli fimbriae and cellulose. These compounds play a crucial role in the initial steps of biofilm formation. For other polyphenolic compounds, such as epigallocatechin gallate, an interference with biofilm formation via the csgD pathway has been proposed [41]. Similar changes in the macrocolonial phenotype were observed for 2″-O-galloyl hyperoside and sundew extracts. It would, therefore, be obvious that similar pathways are influenced here. The role of naphthoquinones and their combinations with flavonoids, however, will be addressed in future studies.

## 3. Materials and Methods

### 3.1. Chemicals

Phytochemicals (purity > 95%): (-)-epigallocatechin gallate (AA blocks, San Diego, CA, USA), quercetin (Sigma-Aldrich Chemie GmbH, Steinheim, Germany), hyperoside, isoquercetin, ellagic acid (Carl Roth GmbH + Co. KG, Karlsruhe, Germany), plumbagin (Acros Organics, Morris Plains, NJ, USA), gallic acid (Merck Schuchardt OHG, Hohenbrunn, Germany); HPLC solvents LC-MS grade: water, acetonitrile, methanol (VWR BDH Chemicals International S.A.S., Rosny-sous-Bios, France).

### 3.2. Plant Material

*D. rotundifolia* and *D. intermedia*, were obtained from Paludimed GmbH (Greifswald, Germany). Plants were cultivated on peat moss turf at Hankhausen Moor in northern Germany (53.26410 N, 8.27239 E) and harvested in July 2020. Immediately after harvesting, plant material was stored at −20 °C and dried in a drying cabinet at 50 °C for 24 h before extraction, then pulverized and sieved (pore size 500 µm). Both above-ground and the below-ground parts of the plants were used. Commercial sundew biomass was obtained via internet and was stored at room temperature. Information of the tested sundew biomasses are summarized in Table 3. Before extraction, the commercial biomass was pulverized and sieved (pore size 500 µm).

### 3.3. Bacteria

Bacterial strains used include three clinically important isolates of *E. coli* (PBIO729, PBIO730, PBIO1986) belonging to pandemic high-risk clonal lineages [19,42]. Detailed information on the strains is summarized in Table 4. The strains were stored in a 20% glycerol solution at −80 °C in cryo-vials. Prior to use, the bacteria were spread on LB agar plates, incubated for 24 h at 37 °C and one colony was picked and suspended in 5 mL of LB medium. The inoculum was incubated at 37 °C with shaking (200 rpm) overnight.

### 3.4. Extraction

#### 3.4.1. Ultrasonic Extraction

300 mg of the powdered sundew biomass was mixed with 10 mL ethanol (99% *V*/*V*) and sonicated at 50 °C for 15 min. Then, the sample was centrifuged at 3500 rpm for 5 min, the clear supernatant was decanted, and ethanol (EtOH) was added again to the residue. In total, the described steps were performed three times and the supernatants were combined. The extract was concentrated under reduced pressure at 40 °C. Last solvent residues were removed by evaporation at room temperature.

#### 3.4.2. Soxhlet Extraction

A Soxhlet extract was prepared for the isolation of the 7-methyl juglone. For this purpose, a conventional Soxhlet system was used in which 2 g of the powdered plant material of *D. rotundifolia* was placed in an extraction vessel, mixed with 2 mL of a tartaric acid solution (0.33 g mL^-1^) and extracted with 150 mL of n-hexane for 2 h.

### 3.5. Solid Phase Extraction

The cartridge (Strata^®^ C18-E 55 µm [70 Å, 20 g/60 mL, Giga Tubes], Phenomenex, Torrance, CA, USA) was equilibrated with 3 × 40 mL of methanol (MeOH), conditioned with 3 × 40 mL of water and then loaded with 150 mg of *Drosera* extract suspended in 5 mL of deionized (DI) water. After loading, a wash step was performed with 40 mL of DI water. To enrich compounds from the sundew extract into various fractions, four different concentrations of MeOH solutions (30%, 42%, 60% and 100%) were prepared for elution. The solutions were acidified with 0.1% anhydrous acetic acid. The extract was eluted with 3 × 40 mL of ascending MeOH concentrations, collecting one eluate for each MeOH concentration. Finally, a wash step was performed with 3 × 40 mL of isopropanol. The flow rate was 2 mL min^-1^ by setting a vacuum. The fractions tested (42%, 60%, and 100% MeOH fractions of *D. rotundifolia* and *D. intermedia*) were concentrated under reduced pressure at 40 °C. Last solvent residues of the 42% MeOH fraction were removed by lyophilization. For the 60% and 100% fractions, final solvent residues were removed by evaporation at room temperature because lyophilization decreased the naphthoquinones concentration.

### 3.6. Size Exclusion Chromatography

Isolation of 7-methyl juglone and 2″-O-galloyl hyperoside was performed by size exclusion chromatography. For this purpose, a glass column was filled with Sephadex^®^ LH-20 (Amersham Biosciences, Uppsala, Sweden) with a length of 55 cm. For 7-methyl juglone, the Soxhlet extract was used because it contained more naphthoquinones than the MeOH extract of *D. rotundifolia*. Isolation of 2″-O-galloyl hyperoside was performed from the 42% MeOH fraction prepared by solid phase extraction from the MeOH extract of *D. rotundifolia* used (see method 3.5). Fractionation allowed, on the one hand, removal of substances that interfere with the isolation step and, on the other hand, enrichment of the 2″-O-galloyl hyperoside. The dried sample (42% MeOH fraction for isolation of 2″-O-galloyl hyperoside and soxhlet extract for isolation of 7-methyl juglone) was dissolved in 0.3 mL of MeOH and added to the column and eluted with MeOH. The eluate was collected in test tubes with a fraction collector (fraction size 1 mL). Fractions were analyzed by HPLC for the target component 2″-O-galloyl hyperoside (extracted from 42% MeOH fraction) or 7-methyl juglone (extracted from soxhlet extract) and corresponding fractions were combined. The solvent of the obtained compounds was evaporated at room temperature.

### 3.7. Determination of Flavonoids

Flavonoid content analysis was performed using two European Pharmacopoeia (Ph.Eur. 9.7, 01/2017:1174) determination methods from the monographs hawthorn leaves with flowers and birch leaves. Both methods are based on photometric analysis of a color complex, the color complex being formed by aluminum in the monograph birch leaves (method A) and by boric acid and oxalic acid in the method for hawthorn leaves with flowers (method B). Flavonoid content analyses were described in detail in Neumann et al. [43].

### 3.8. High Performance Liquid Chromatography

For comparison, an high performance liquid chromatography (HPLC) method based on Zehl et al. was developed, which allowed quantification of four flavonoids (hyperoside, isoquercetin, quercetin and 2″-O-galloyl hyperoside), as well as two naphthoquinone compounds (plumbagin and 7-methyl juglone) [7]. A Shimadzu chromatography system (LC-20AD, Shimadzu Scientific Instruments, Nakagyo-ku, Kyōto, Japan) with a photodiode array detector (PDA) was used. A Luna^®^ C18 column (100 Å, 250 × 4.6 mm) from Phenomenex^®^ (Torrance, CA, USA) served as the stationary phase. The mobile phase consisted of a water-acetonitrile (ACN) mixture acidified with 0.1% acetic acid (glacial, analytical grade). The initial composition of the mobile phase was 14% ACN and was changed linearly to 16% over 15 min. After a 4 min plateau, the concentration increases to 40% ACN, occurring within 0.5 min, and from there a further increase comes within 9.5 min to 80% ACN for 2 minutes. Subsequently, the initial concentration of 14% ACN was adjusted. The total time was 35 min, at a flow rate of 1.2 mL min^−1^. From the previously dissolved in MeOH and filtered samples (0.22 mm polytetrafluorethylene [PTFE] filter, Brand GmbH + Co. KG, Wertheim, Germany), 10 µL were injected. UV detection was performed at 254 nm. This method was used to quantify the substances hyperoside, isoquercetin, quercetin, 2″-O-galloyl hyperoside, ellagic acid, gallic acid, 7-methyl juglone and plumbagin. Calibration lines were determined eight times, in a concentration range from 10 to 100 µg mL^−1^ for gallic acid, ellagic acid, hyperoside, isoquercetin and quercetin. For plumbagin, the calibration lines were determined in a concentration range from 10–200 µg mL^−1^ and for 7-methyl juglone and 2″-O-galloyl hyperoside from 10 to 100 µg mL^−1^. Calibration lines were determined for plumbagin, 7-methyl juglone and 2″-O-galloyl hyperoside five times. Information on the quantified substances is summarized in Table 5.

### 3.9. LC-MS Analysis

An LCMS-8030 triple quadrupole mass spectrometer (Shimadzu Scientific Instruments, Columbia, SC, USA) with an electrospray ionization (ESI) source was used for mass spectrometric analysis. Samples were dissolved in MeOH and adjusted to a concentration of 10 µg mL^−1^.

The following data refer to the settings for the identification of 2″-O-galloyl hyperoside. A mixture of water and MeOH containing 0.1% acetic acid (50:50) with a flow rate of 0.6 mL min^−1^ was used as mobile phase. The following ESI interface conditions were set: nebulization gas flow at 3 L min^−1^, desolvation line temperature at 250 °C, heating block temperature at 400 °C and drying gas flow at 15 L min^−1^. Nitrogen served as nebulizer and argon as collision gas. The ESI ion source was used in negative and positive modes. A scan in a range *m*/*z* 100 up to 1000 was performed first, followed by MRM (multiple reaction monitoring) optimization, in which the five most intense MRM transitions were analyzed. For the identification of 7-methyl juglone, the ESI interface conditions were set as follows: nebulization gas flow at 3 L min^−1^, desolvation line temperature at 300 °C, heating block temperature at 500 °C and drying gas flow at 10 L min^−1^. The other conditions were the same as for 2″-O-galloyl hyperoside.

### 3.10. Minimum Inhibitory Concentration

A serial dilution of sundew extracts (1000 µg mL^−1^, 400 µg mL^−1^, 300 µg mL^−1^, 200 µg mL^−1^, 150 µg mL^−1^, 100 µg mL^−1^, 50 µg mL^−1^, 25 µg mL^−1^) with EtOH was tested. Amounts of 100 to 10 µL of a stock solution 1 (10 mg mL^−1^) and 50 to 25 µL of a stock solution 2 (1 mg mL^−1^) were pipetted into the wells of a microtiter plate (96-wells, sterile F, Carl Roth, Karlsruhe, Germany). After the solvent was evaporated, 198 µL of Mueller Hinton II medium was pipetted into each well and added with 2 µL of bacterial suspension adjusted to an OD of 0.5. Growth control, positive control with chloramphenicol and solution controls with EtOH were performed in parallel. After 24 h incubation at 37 °C, the MIC value was evaluated visually. The test was conducted in triplicates.

### 3.11. Biofilm Assay

**Figure 9:**
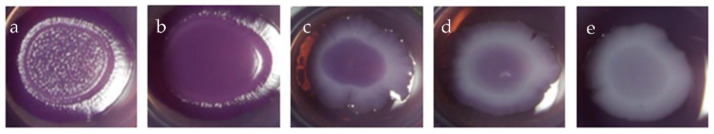
Staining of adhesive matrix components cellulose and curli fimbriae (**a**) and cellulose only (**b**); inhibition of cellulose and curli fimbriae formation at different intensities (**c**–**e**).

Different concentrations (400 µg mL^−1^, 300 µg mL^−1^, 200 µg mL^−1^, 100 µg mL^−1^, 150 µg mL^−1^, 50 µg mL^−1^, 25 µg mL^−1^, 12.5 µg mL^−1^) of the extracts and pure compounds were pipetted into a 24-well plate, the solvent was evaporated at room temperature, and 1 mL of liquefied Congo red agar (color solution: 50 mg Congo red, 25 mg Coomassie brilliant blue R250, 17.5 mL EtOH, 2.5 mL Aq. Bidest.; 2% of the color solution added to the agar: 10 g Bacto Tryptone, 5 g Bacto Yeast Extract, 18 g span agar, 1 L water) was pipetted into each well. After the agar had cooled down, 5 µL of a bacterial suspension with an OD of 0.5 was placed on the agar. After the bacterial suspension was dried on the plate, the plate was sealed with Parafilm^®^, incubated for 48 h at 28 °C. The production of curli fibers and cellulose was examined visually, a decolorization of the surface of the macrocolony signaled an inhibition of the matrix component studied (see Figure 9). The assay was performed in triplicate. For a detailed description of the assay, see Stepanov et al. [44].

### 3.12. Software

Shimadzu LabSolutions version 5.85 was used for LC and MS data analysis. Graphpad Prism version 9.4.1 was used for the creation of the graphs and the statistical analysis.

## 4. Conclusions

In the present study, the inhibitory effects of extracts and components of extracts (naphthoquinones, flavonoids and ellagic acid derivatives) from two European sundew species (*D. rotundifolia* and *D. intermedia*) and commercial sundew biomass on the formation of biofilm matrix components cellulose and curli fimbria against three different multi-resistant *E. coli* strains were tested. The minimum biofilm inhibitory concentration of the European species was significantly lower than of the commercial sundew biomass. Quercetin, 7-methyl juglone, ellagic acid and 2″-O-galloyl hyperoside had biofilm inhibitory effects, with 2″-O-galloyl hyperoside showing the most potent effect. The extract showed to be most effective against all three multidrug-resistant *E. coli* strains, compared to the single compounds, which may be due to additive combinatory or synergistic effects. In conclusion, the tested European sundew species *D. rotundifolia* and *D. intermedia* are preferable as potential antibiofilm agents, compared to the purchased biomasses, and they should be applied in the form of a total extract instead of isolating single compounds. In general, it can be said that this study indicates a promising new indication area, the therapy of urinary tract infections, for the pharmaceutical medicinal plant sundew. The inflationary prescription of antibiotics for the therapy of harmless urinary tract infections led to the proliferation of multi-resistant germs, such as *E. coli*. Summarized, sundew showed a biofilm inhibitory effect. This could be mainly due to the flavonoids. Phytopharmaceuticals are already integrated in the pharmaceutical market as an adjunctive therapy for urinary tract infections. This study could indicate another possible indication for sundew, the therapy of multidrug-resistant germs. In particular, in urinary tract diseases, the frequent use of antibiotics for the therapy of harmless urinary tract infections led to the spread of multi-resistant germs, such as *E. coli.* Therefore, the search for innovative therapy options is relevant.

## Figures and Tables

**Table 1:** Conditions for multiple reaction monitoring of isolated compounds using a triple quadrupole mass spectrometer.

Compound	MRM (ESI +)	Q1 pre Bias (eV)	Collisions Energy (eV)	Q3 pre Bias (eV)	Compound
7-methyl juglone	188.9 → 161.15	−12	−18	−11	7-methyl juglone
	188.9 → 114.95	−12	−28	−12	
	188.9 → 105.10	−12	−23	−18	
	188.9 → 77.05	−24	−45	−15	
	188.9 → 157.05	−25	−9	−15	
plumbagin	188.9 → 121.05	−12	−22	−12	plumbagin
	188.9 → 144.95	−12	−31	−19	
	188.9 → 161.15	−12	−18	−11	
	188.9 → 65.20	−12	−43	−11	
	188.9 → 105.15	−12	−23	−18	

**Table 2:** Conditions for multiple reaction monitoring of isolated compounds using a triple quadrupole mass spectrometer.

Compound	MRM (ESI +)	MRM (ESI −)	Q1 pre Bias (V)	Collisions Energy (V)	Q3 pre Bias (V)
2″-O-galloyl hyperoside	617.10 → 315.05		−30	−14	−22
	617.10 → 153.00		−30	−33	−15
	617.10 → 302.95		−28	−18	−14
	617.10 → 297.00		−20	−18	−20
	617.10 → 233.10		−22	−27	−15
		615.30 → 300.90	28	24	24
		615.30 → 150.95	28	53	10
		615.30 → 179.00	28	45	10
		615.30 → 312.90	28	23	10
		615.30 → 121.10	28	49	12

**Table 3:** Overview of the purchased sundew biomass samples and sundew biomass samples cultivated in Germany.

Sample Name	Product Description	Company
*D. rotundifolia*	harvested fresh biomass, whole plant	Paludimed GmbH (Greifswald, Germany)
*D. intermedia*	harvested fresh biomass, whole plant	Paludimed GmbH (Greifswald, Germany)
*D. longifolia* (KG)	declared as *Drosera longifolia*, cut biomass	Kräutergarten (Munich, Germany)
*D. longifolia* (Ga)	declared as *Drosera longifolia*, cut biomass	Alfred Galke GmbH (Bad Grund, Germany)
*Drosera* sp. (Pini)	declared as *Drosera* sp., cut biomass	Pinisan Laboratorios (Madrid, Spain)
D. planta trit. (Plam)	declared as Drosera planta trit., cut biomass	Plameca (Barcelona, Spain)

**Table 4:** Characteristics of the *E. coli* strains used in this study.

Strain/ Data Number	Host	Origin	Toxin-Antitoxin System	Other Epigenomic Resistance Genes	Genome Accession Numbers/	CTX-M Type, Sequence Type
*E. coli*/ IMT17433 PBIO729	dog (*C. lupus familiaris*)	urinary tract infection	pemI/K, vagC/D, hok/sok	blaTEM-1, blaOXA-1, tet(A), tet(R), aadA, aac(6′)-ib-cr	ERR163891	CTX-M-15 ST131
*E. coli*/ IMT16316 PBIO730	blackbird (*T. merula*)	feces	pemI/K, vagC/D, srnB/C,	tet(A), tet(R), sul1, sul2, strA, strB, aadA, aac(3)-II mph(A), mrx, mphR, dhfrVII	ERR163879	CTX-M-15 ST648
*E. coli*/ IMT17887 PBIO1986	horse (*E. ferus* *caballus*)	soft tissue/wound infection	pemI/K, vagC/D, srnB/C	tet(A), tet(R), sul1, sul2, strA, strB, aadA, mph(A), mphR, dhfrVII	ERR163883	CTX-M-15 ST648

**Table 5:** Linearity of calibration, limits of detection (LOD) and limits of quantification (LOQ) for six commercially available compounds (hyperoside, isoquercetin, quercetin, gallic acid, ellagic acid and plumbagin) and two isolated compounds (2″-O-galloyl hyperoside and 7-methyl juglone) in the extract of *Drosera* species.

Compound	RT	Linear Range (µg mL^−1^)	R^2^	Calibration Equation *	LOD ** (ng mL^−1^)	LOQ ** (ng mL^−1^)
hyperoside	17.35	10–100	0.9998	y = 1,999,316x − 28,941	0.62	1.73
isoquercetin	18.7	10–100	0.9999	y = 1,890,097x − 15,975	0.65	1.77
quercetin	24.96	10–100	0.9999	y = 2,740,278x − 11,469	0.30	0.82
2″-O-galloyl hyperoside	19.43	10–100	0.9999	y = 2,148,206x − 10,079	0.46	1.33
gallic acid	3.11	10–100	0.9999	y = 1,327,487x + 15,200	3.90	8.15
ellagic acid	15.7	10–100	0.9905	y = 4,978,062x − 570,036	1.51	4.51
plumbagin	28.4	10–200	0.9984	y = 2,893,059x + 27,732	0.14	0.30
7-methyl juglone	28.1	10–100	0.9996	y = 2,042,891x + 7477	0.27	0.73

* Peak area vs. concentration, ** Signal-to-noise.

## Supplementary Materials

The following supporting information can be downloaded at: https://www.mdpi.com/article/10.3390/ijms232213720/s1.
