# Supplementary material for: Antibiofilm Activity of Sundew Species against Multidrug-Resistant Escherichia coli Strains
Source: Int J Mol Sci. 2022 Nov 8;23(22):13720. doi: 10.3390/ijms232213720 (PMC9697453; doi:10.3390/ijms232213720)
Supplement: Supplementary file 1 [file ijms-23-13720-s001.zip › ijms-2000497-supplementary.pdf]

Table S1. Minimum inhibitory concentration (MIC) of the EtOH extracts and MeOH fractions (42%, 60%, 100%) of *D. rotundifolia* and *D. intermedia*, also single compounds (hyperoside, isoquercetin, quercetin, 2’’-O-galloyl hyperoside, ellagic acid, gallic acid, plumbagin, 7-methyl juglone) against three multidrug-resistant *E. coli* strains, n = 3.

Sample		Tested bacteria		
		E. coli		
		PBIO729	PBIO730	PBIO1986
		MIC µg mL^{-1} (SD)		
D. rotundifolia				
	EtOH extract	> 1000	> 1000	> 1000
	42% MeOH fraction	> 1000	> 1000	> 1000
	60% MeOH fraction	> 1000	> 1000	> 1000
	100% MeOH fraction	> 1000	> 1000	> 1000
D. intermedia				
	EtOH extract	> 1000	> 1000	> 1000
	42% MeOH fraction	> 1000	> 1000	> 1000
	60% MeOH fraction	> 1000	> 1000	> 1000
	100% MeOH fraction	367 (± 115.47)	700 (± 692.82)	700 (± 692.82)
D. longifolia (KG)	EtOH extract	> 1000	> 1000	> 1000
D. longifolia (Ga)	EtOH extract	> 1000	> 1000	> 1000
Drosera sp. (Pini)	EtOH extract	> 1000	> 1000	> 1000
D. planta trit.(Plam)	EtOH extract	> 1000	> 1000	> 1000
single compounds				
	Hyperoside	> 1000	> 1000	> 1000
	2’’-O-galloyl hyperoside	> 1000	> 1000	> 1000
	Quercetin	> 1000	> 1000	> 1000
	Isoquercetin	> 1000	> 1000	> 1000
	Plumbagin	104 (± 36.08)	167 (± 72.17)	208 (± 72.17)
	7-methyl juglone	250 (± 216.51)	292 (± 190.94)	333 (± 144.34)
	gallic acid	> 1000	> 1000	> 1000
	ellagic acid	> 1000	> 1000	> 1000
